# Foundational Statistical Principles in Medical Research: Sensitivity, Specificity, Positive Predictive Value, and Negative Predictive Value

**DOI:** 10.3390/medicina57050503

**Published:** 2021-05-16

**Authors:** Thomas F. Monaghan, Syed N. Rahman, Christina W. Agudelo, Alan J. Wein, Jason M. Lazar, Karel Everaert, Roger R. Dmochowski

**Affiliations:** 1 Department of Urology, University of Texas Southwestern Medical Center, Dallas, TX 75390, USA; 2 Department of Urology, Yale University School of Medicine, New Haven, CT 06520, USA; syed.rahman@downstate.edu; 3 Division of Cardiovascular Medicine, Department of Medicine, SUNY Downstate Health Sciences University, Brooklyn, NY 11203, USA; christina.agudelo@downstate.edu (C.W.A.); Jason.lazar@downstate.edu (J.M.L.); 4 Division of Urology, Perelman School of Medicine at the University of Pennsylvania, Philadelphia, PA 19104, USA; alan.wein@pennmedicine.upenn.edu; 5 Department of Human Structure and Repair, Ghent University, 9000 Ghent, Belgium; karel.everaert@uzgent.be; 6 Department of Urological Surgery, Vanderbilt University Medical Center, Nashville, TN 37232, USA; roger.dmochowski@vumc.org

**Keywords:** basics, biostatistics, diagnosis, fundamentals, introduction, methodology, overview, screening, statistics, tutorial

## Abstract

Sensitivity, which denotes the proportion of subjects correctly given a positive assignment out of all subjects who are actually positive for the outcome, indicates how well a test can classify subjects who truly have the outcome of interest. Specificity, which denotes the proportion of subjects correctly given a negative assignment out of all subjects who are actually negative for the outcome, indicates how well a test can classify subjects who truly do not have the outcome of interest. Positive predictive value reflects the proportion of subjects with a positive test result who truly have the outcome of interest. Negative predictive value reflects the proportion of subjects with a negative test result who truly do not have the outcome of interest. Sensitivity and specificity are inversely related, wherein one increases as the other decreases, but are generally considered stable for a given test, whereas positive and negative predictive values do inherently vary with pre-test probability (e.g., changes in population disease prevalence). This article will further detail the concepts of sensitivity, specificity, and predictive values using a recent real-world example from the medical literature.

## 1. Introduction

Diagnostic testing is used to differentiate between individuals with and without a disease or other condition of interest. Most diseases have ‘gold standard,’ or benchmark test, against which alternative diagnostic tests can be assessed [1]. Gold standard tests take many shapes and forms, but the discussion of what is ‘gold’ generally centers on diagnostic yield (i.e., how well the test correctly identifies diseased subjects as positive and non-diseased subjects as negative), rather than what is ‘gold’ in terms of availability, cost-effectiveness, or nonmaleficence [1]. Thus, a gold standard test may very well be too resource-consuming, costly, or invasive to be practical for widespread use. Such scenarios require an alternative diagnostic test that is better able to balance diagnostic yield with pragmatism.

## 2. Sensitivity and Specificity

Sensitivity and specificity are the two statistical measures most commonly used to assess the performance of an alternative test against the gold standard. We will approach sensitivity and specificity using a recent real-world example from the medical literature. We will also use these data in subsequent sections of this article to explore the concepts of positive predictive value and negative predictive value.

Prostate-specific antigen (PSA) is a protein produced by cells of the prostate gland. The PSA test measures the level of PSA in a man’s blood and is widely used to screen for prostate cancer. A newer, related parameter, PSA density, is computed as the serum PSA level (in ng/mL) divided by the volume of the prostate gland (in cubic centimeters (cc)). Unlike the PSA test itself, PSA density accounts for the fact that men with larger prostates generally have higher levels of PSA under normal baseline physiologic conditions (i.e., in the absence of prostate cancer) [2]. Accordingly, consideration of PSA density is increasingly favored over PSA alone for assessing prostate cancer risk [3,4,5]. 

In a recent study published in the Journal of Urology, Aminsharifi et al. sought to determine the diagnostic utility of PSA density (i.e., an ‘alternative test’) in detecting clinically significant prostate cancer in men with elevated PSA who underwent prostate biopsy [6]. The authors obtained institutional review board approval to retrospectively review data collected on 4109 men who underwent transrectal ultrasound-guided prostate biopsy at their institution from January 2002 through May 2017. PSA was measured prior to biopsy, and prostate volume was determined on transrectal ultrasound at the time of biopsy. Only first available biopsy results were included from men with repeat biopsies. After excluding subjects with a PSA outside of the prespecified range (<4 or >10 ng/mL), or with missing data, a total of 2162 men were included in the final study cohort. 

For simplicity, we will assume that prostate biopsy results (‘gold standard’) correctly determined the true disease status (i.e., presence versus absence of clinically significant prostate cancer) in all cases. Thus, there exist four basic combinations of ‘assignment status’ (i.e., cancer status according to PSA density) and ‘actual outcome’ (i.e., cancer status according to gold standard prostate biopsy) by which all 2162 subjects may be categorized (Table 1). In this scenario, ‘true positive’ denotes subjects with an actual positive outcome who were correctly given a positive assignment (i.e., PSA density test-positive, biopsy-positive for prostate cancer). ‘True negative’ indicates subjects with an actual negative outcome who were correctly given a negative assignment (i.e., PSA density test-negative, biopsy-negative). ‘False positive’ denotes subjects with an actual negative outcome who were incorrectly given a positive assignment (i.e., PSA density test-positive, biopsy-negative). ‘False negative’ indicates subjects with an actual positive outcome who were incorrectly given a negative assignment (i.e., PSA density test-negative, biopsy-positive). 

The optimal PSA density cutoff for biopsy decision-making, based a priori on a negative predictive value (defined later in this article) of 95%, was determined to be 0.08 ng/mL/cc. In other words, if the study were to have been performed prospectively, subjects with a PSA density ≥ 0.08 ng/mL/cc would undergo prostate biopsy while those with a PSA density < 0.08 ng/mL/cc would avoid biopsy. Using a PSA density cutoff of 0.08 ng/mL/cc, Aminsharifi et al. identified 489 true positives, 263 true negatives, 1400 false positives, and 10 false negative subjects (Table 2).

Sensitivity, or true positive rate, quantifies how well a test identifies true positives (i.e., how well a test can classify subjects who truly have the condition of interest). Stated alternatively, sensitivity measures the proportion of subjects with an actual positive outcome (i.e., true positives + false negatives) who are correctly given a positive assignment (i.e., true positives only).

$$Sensitivity=\frac{\#\;Correctly\;Identified\;as\;Affected}{\#\;Total\;Affected}=\frac{\#\;True\;Positives}{\left(\#\;True\;Positives+\#\;False\;Negatives\right)}$$

In our working example, 489 subjects were identified as true positives and 10 subjects were false negatives, corresponding to a sensitivity of 98% (489/(489 + 10)). In other words, ‘98% sensitivity’ indicates that 489 out of 499 (98%) subjects with clinically significant prostate cancer were correctly identified as positive using a PSA density cutoff ≥0.08 ng/mL/cc. Thus, in the present study, PSA density ≥0.08 ng/mL/cc was found to correctly identify 98% of all subjects with clinically significant prostate cancer. 

Specificity, or true negative rate, quantifies how well a test identifies true negatives (i.e., how well a test can classify subjects who truly do not have the condition of interest). Stated alternatively, specificity measures the proportion of subjects with an actual negative outcome (i.e., true negatives + false positives) who are correctly given a negative assignment (i.e., true negatives only).

$$Specificity=\frac{\#\;Correctly\;Identified\;as\;Unaffected}{\#\;Total\;Unaffected}=\frac{\#\;True\;Negatives}{\left(\#\;True\;Negatives+\#\;False\;Positives\right)}$$

In our working example, 263 subjects were identified as true negatives and 1400 subjects were false positives, corresponding to a specificity of 16% (263/(1400 + 263)). In other words, ‘16% specificity’ indicates that 263 out of 1663 (16%) subjects without clinically significant prostate cancer were correctly identified as negative using a PSA density cutoff ≥0.08 ng/mL/cc. Thus, in the present study, a negative PSA density (<0.08 ng/mL/cc) was found to correctly identify 16% of all subjects without clinically significant prostate cancer.

## 3. Sensitivity vs. Specificity

Recall that, using a PSA density cutoff of ≥0.08 ng/mL/cc as the threshold for prostate biopsy, very few truly diseased subjects (2%) would be found to have a PSA density less than 0.08 ng/mL/cc (based on 98% sensitivity). Conversely, however, many truly non-diseased subjects (84%) would, in fact, have a PSA density greater than 0.08 ng/mL/cc (based on 16% specificity). This allows us to think intuitively about what we might expect to happen if we lowered the PSA density threshold for prostate biopsy from 0.08 ng/mL/cc to 0.05 ng/mL/cc, or, instead, if we raised the threshold from 0.08 ng/mL/cc to 0.15 ng/mL/cc.

If the threshold were decreased to ≥0.05 ng/mL/cc, it stands to reason that even fewer truly diseased subjects would fall below the cutoff for prostate biopsy (i.e., fewer false negatives, corresponding to more true positives, or higher sensitivity). However, at this lower PSA density threshold, we would also expect even more truly non-diseased subjects to have a PSA density above the cutoff for prostate biopsy (i.e., more false positives, corresponding to fewer true negatives, or lower specificity). Indeed, Aminsharifi et al. reported a 99.6% (vs. 98%) sensitivity and 3% (vs. 16%) specificity for a PSA density threshold of ≥0.05 ng/mL/cc instead of ≥0.08 ng/mL/cc. 

On the other hand, if the threshold were increased to ≥0.15 ng/mL/cc, it stands to reason that more truly diseased subjects would fall below the cutoff for prostate biopsy (i.e., more false negatives, corresponding to fewer true positives, or lower sensitivity). However, at this higher PSA density threshold, we would also expect fewer truly non-diseased subjects to have a PSA density above the cutoff for prostate biopsy (i.e., fewer false positives, corresponding to more true negatives, or higher specificity). Consistently, Aminsharifi et al. reported a 72% sensitivity (versus 98%) and 57% (versus 16%) specificity for a PSA density threshold of ≥0.15 ng/mL/cc instead of ≥0.08 ng/mL/cc.

Taken together, our understanding of changes in sensitivity and specificity at different cutoff values for a given binary classification test underscores the delicate balance between these two common statistical measures of test performance. It is important to recognize that sensitivity and specificity will always be inversely related (i.e., one increases as the other decreases). Namely, each discrete point along the continuum of potential cutoff values for a given diagnostic test (e.g., PSA density 0.08 ng/mL/cc versus 0.05 or 0.15 ng/mL/cc) will be accompanied by an intrinsic sensitivity and specificity, with higher sensitivity occurring at relatively lower cut points, and higher specificity achieved at higher cut points. Therefore, in practice, the process of selecting a discrete threshold value for a given test must carefully weigh the relative importance of a high true positive rate versus a high true negative rate and, by extension, the consequences of false negative and false positive results for the particular test at hand [7]. Note that, while sensitivity and specificity are basic statistical principles for assessing the performance of diagnostic tests, they also form the basis for understanding misclassification in more complex statistical analyses. For example, there exist a number of methods such as resampling techniques and feature selection to reduce the risk of misclassification in machine learning models [8].

## 4. Positive Predictive Value and Negative Predictive Value

Sensitivity and specificity are highly relevant statistical parameters for assessing the performance of a diagnostic test. However, in real-world practice, rather than knowing the proportion of diseased patients who will test positive (or non-diseased patients who will test negative), it is often more meaningful to predict whether a particular person will truly have the disease based on a positive or negative test result. To this end, positive predictive value and negative predictive value reflect the proportion of positive and negative results that are true positives and true negatives, respectively. In other words, positive predictive value answers the question, ‘if I have a positive test, what is the probability that I actually have the disease?’ Conversely, negative predictive value answers the question, ‘if I have a negative test, what is the probability that I actually don’t have the disease?’

$$Positive\;Predictive\;Value=\frac{\#\;True\;Positives}{\#\;Positive\;Calls}=\frac{\#\;True\;Positives}{\left(\#\;True\;Positives+\#\;False\;Positives\right)}\;Negative\;Predictive\;Value=\frac{\#\;True\;Negatives}{\#\;Negative\;Calls}=\frac{\#\;True\;Negatives}{\left(\#\;True\;Negatives+\#\;False\;Negatives\right)}$$

Returning to the results from Aminsharifi et al., the positive predictive value and negative predictive value using a PSA density cutoff of ≥0.08 ng/mL/cc were found to be 26% (489/(489 + 1400)) and 96% (263/(263 + 10)), respectively. In other words, 26% of subjects with a PSA density ≥0.08 ng/mL/cc truly had clinically significant prostate cancer, and 96% of subjects with a PSA density <0.08 ng/mL/cc truly did not have clinically significant prostate cancer. Thus, if the present study had been performed prospectively, 26% of subjects warranting prostate biopsy based on a PSA density ≥0.08 ng/mL/cc would have truly had clinically significant prostate cancer, while 96% of subjects who would have been excluded from prostate biopsy (PSA density <0.08 ng/mL/cc) would truly not have had clinically significant prostate cancer.

## 5. Predictive Values vs. Pretest Probability

While sensitivity and specificity are generally considered stable features of a given test, positive and negative predictive values depend on the pre-test probability (i.e., probability of the presence of the disease before a diagnostic test), which is determined by baseline risk factors such as disease prevalence [9]. To better understand this, suppose we apply the same diagnostic test (PSA density), using the same cutoff value (≥0.08 ng/mL/cc) to assess the same outcome (clinically significant prostate cancer), but now in a different population. As with the original study sample, we will also assess data from 2162 men in our new population. However, compared to our original population sample, wherein 499 out of 2162 subjects were found to truly have clinically significant prostate cancer, the prevalence of clinically significant prostate cancer is either much lower or much higher in our new population. 

We understand that a change in disease prevalence means that fewer or more study subjects will have the disease in the absolute sense (e.g., 499/2162 versus only 10/2162 or 1000/2162). However, because we are using the exact same test and cutoff value to assess the exact same outcome, would there be any reason to expect a change in the proportion of diseased subjects who are successfully identified by the diagnostic test (i.e., sensitivity)? Likewise, would there be any reason to expect a change in the proportion of non-diseased subjects who are successfully identified as negative by the diagnostic test (i.e., specificity)? The answer to both questions is no. Whether 10 or 499 or 1000 out of 2162 subjects have clinically significant prostate cancer, we should expect no difference in the percentage of diseased subjects who are correctly identified by the PSA density test as diseased (i.e., true positives), nor any difference in the percentage of non-diseased subjects correctly identified by the PSA density test as non-diseased (i.e., true negatives). In other words, neither sensitivity nor specificity of PSA density ≥0.08 ng/mL/cc for clinically significant prostate cancer would be expected to change when this new population is assessed.

Now suppose that you work at a busy urology practice in the same town from which all of these new 2162 subjects were recruited. A nervous patient walks through your office door and asks for results of the labs you had drawn the week prior. His PSA is elevated at 5 ng/mL (reference range: <4 ng/mL) and, based on the results from Aminsharifi et al., his PSA density of 0.11 ng/mL/cc is also elevated (≥0.08 ng/mL/cc) [6,10]. Owing to his lab results, you explain that he warrants further workup for prostate cancer, and recommend that he schedule a prostate biopsy. “But Doctor,” he says, “what are the chances that I actually have prostate cancer?” You pause. Would you be more confident in that positive test result if only 1-in-1,000,000 similar patients in your practice who had a PSA density >0.08 ng/mL/cc turned out to truly have clinically significant prostate cancer, or if 999,999-in-1,000,000 similar patients who had a PSA density >0.08 ng/mL/cc turned out to truly have clinically significant prostate cancer? Intuitively, if most of your patients, even despite a positive test result, were ultimately found to be negative for clinically significant prostate cancer, you might be inclined to reassure this man: “don’t worry, we see these positive results all the time but they almost never amount to anything—not something to lose sleep over.” Conversely, if the vast majority of patients presenting with similar laboratory results did ultimately prove to have clinically significant prostate cancer, there may even be a heightened sense of urgency in your recommendation for this man to move forward with a prostate biopsy. 

Your next patient also has a PSA of 5 ng/mL (reference range: <4 ng/mL), but his PSA density is 0.03 ng/mL—below the 0.08 ng/mL threshold from Aminsharifi et al. [6,10]. “I know high PSA is bad,” he says, “but you’re saying that it might just be because I have a big prostate—does this mean that I don’t have cancer?” You pause again. Would you have more confidence in that negative PSA density test result if 99% of similar patients in your practice who had a PSA density <0.08 ng/mL/cc actually did not turn out to have prostate cancer, or if 99% of similar patients who had a PSA density <0.08 ng/mL/cc actually did turn out to have clinically significant prostate cancer? It stands to reason that most clinicians would be inclined to trust this negative test result if most of their patients who tested negative did, in fact, ultimately prove negative on further work-up, but have less trust in this result if most of their patients who tested negative were nevertheless ultimately found to have clinically significant prostate cancer. 

Sensitivity and specificity should be considered inherently stable measures of test performance for all intents and purposes [11,12]. (In practice, some study design and population factors may indirectly cause sensitivity and specificity to vary with disease prevalence, but this advanced topic is beyond the scope of the present article [13].) Both positive predictive value and negative predictive value, in contrast to sensitivity and specificity, will change with disease prevalence. The more common the disease, the more sure we can be that a positive test really indicates disease, and the less sure that a negative result indicates no disease (i.e., positive predictive value will increase with increased disease prevalence, and negative predictive value will decrease with increased disease prevalence) [14]. Conversely, the rarer the disease, the more sure we can be that a negative result indicates no disease, and the less sure we can be that a positive test really indicates disease (i.e., negative predictive value will increase with decreased disease prevalence, and positive predictive value will decrease with decreased disease prevalence) [14].

We can use the actual sensitivity (98%) and specificity (16%) values reported by Aminsharifi et al. to further illustrate the stability of these measures for a given cutoff value (≥0.08 ng/mL/cc) and sample size (*n* = 2162) despite changes in disease prevalence (and corresponding changes in positive predictive value and negative predictive value) (Table 3). Quantitively, as noted by Altman and Bland, the predictive values can be computed if we know sensitivity, specificity, and disease prevalence as follows [14]:

$$Positive\;Predictive\;Value=\frac{Sensitivity\times Prevalence}{Sensitivity\times Prevalence+\left(1-Specificity\right)\times \left(1-Prevalence\right)}$$

$$Negative\;Predictive\;Value=\frac{Specificity\times \left(1-Prevalence\right)}{\left(1-Sensitivity\right)\times Prevalence+Specificity\times \left(1-Prevalence\right)}$$

## 6. Summary

Sensitivity and specificity measure how well a test classifies subjects who truly have/do not have the outcome of interest, respectively. In practice, the process of selecting a discrete threshold value for a given test must carefully weigh the relative importance of a high true positive rate versus a high true negative rate and, by extension, the consequences of false negative and false positive results for the particular test at hand. Positive and negative predictive value reflect the proportion of positive and negative test results, respectively, that are truly positive and truly negative. In contrast to sensitivity and specificity, which are generally considered inherently stable for a given diagnostic test, positive predictive value and negative predictive value are highly dependent on pre-test probability, wherein positive predictive values increase with increased disease prevalence, and negative predictive values increase with decreased disease prevalence.

## Figures and Tables

**Table 1:** Standard 2 × 2 contingency table depicting possible outcomes of a binary classification test.

		**Actual Status**		
		Disease Positive	Disease Negative	
**Assignment Status**	Test Positive	TP	FP	PPV =TP/(TP + FP)
	Test Negative	FN	TN	NPV =TN/(TN + FN)
		Sensitivity =TP/(TP + FN)	Specificity =TN/(TN + FP)	

Note: Abbreviations—FN, false negative; FP, false positive; NPV, negative predictive value; PPV, positive predictive value; TN, true negative; TP, true positive.

**Table 2:** Prostate cancer assignment status based on prostate-specific antigen density (‘alternative test’) versus prostate biopsy (‘gold standard’).

	**Disease Positive**	**Disease Negative**	**Row Total**
PSAD Positive	489	1400	1889
PSAD Negative	10	263	273
Column Total	499	1663	

Note: Following the analysis from Aminsharifi et al., which retrospectively established a PSAD cutoff of ≥0.08 ng/mL/cc as the optimal threshold for proceeding with prostate biopsy in the diagnosis of clinically significant prostate cancer [6], biopsy would have been performed in 489 subjects with (true positives) and 1400 subjects without (false positives) clinically significant prostate cancer. Additionally, biopsy would have been avoided in 263 subjects without clinically significant prostate cancer (true negatives), as well as 10 subjects with clinically significant prostate cancer (false negatives). Abbreviations—PSAD, prostate-specific antigen density.

**Table 3:** Sensitivity, specificity, positive predictive value, and negative predictive value of PSAD ≥ 0.08 ng/mL/cc for clinically significant prostate cancer in 2162 subjects with varying disease prevalence.

**Hypothetical Population (*n* = 2162) with 50% Disease Prevalence**				
	**Disease Positive**	**Disease Negative**	**Row Total**	**Predictive Values**
PSAD Positive	1059	908	1967	(↑) PPV = 1059/1967 (54%)
PSAD Negative	22	173	195	(↓) NPV = 173/195 (89%)
Column Total	1081	1081		
	SN = 1059/1081 (98%)	SP = 173/1081 (16%)		
**Actual Study Population (23% Disease Prevalence, 98% Sensitivity, 16% Specificity) (*n* = 2162)**				
	**Disease Positive**	**Disease Negative**	**Row Total**	**Predictive Values**
PSAD Positive	489	1400	1889	PPV = 489/1889 (26%)
PSAD Negative	10	263	273	NPV = 263/273 (96%)
Column Total	499	1663		
	SN = 489/499 (98%)	SP = 263/1663 (16%)		
**Hypothetical Population (*n* = 2162) with 10% Disease Prevalence**				
	**Disease Positive**	**Disease Negative**	**Row Total**	**Predictive Values**
PSAD Postive	212	1635	1847	(↓) PPV = 212/1847 (11%)
PSAD Negative	4	311	315	(↑) NPV = 311/315 (99%)
Column Total	216	1946		
	SN = 212/216 (98%)	SP = 311/1946 (16%)		

Note: Abbreviations—NPV, negative predictive value; PPV, positive predictive value; PSAD, prostate-specific antigen density; SN, sensitivity; SP, specificity.

## Data Availability

Not applicable.
